# Shortages of Medicines to Treat COVID-19 Symptoms during the First Wave and Fourth Wave: Analysis of Notifications Reported to Registers in Austria, Italy, and Spain

**DOI:** 10.3390/pharmacy11040120

**Published:** 2023-07-21

**Authors:** Diana Ivonne Rodríguez Sánchez, Sabine Vogler

**Affiliations:** 1 Faculty of Health Sciences, Jagiellonian University, 31-126 Krakow, Poland; diana.ivonnerdz@gmail.com; 2 WHO Collaborating Centre for Pharmaceutical Pricing and Reimbursement Policies, Pharmacoeconomics Department, Gesundheit Österreich (GÖG/Austrian National Public Health Institute), 1010 Vienna, Austria; 3 Department of Health Care Management, Technische Universität Berlin, 10623 Berlin, Germany

**Keywords:** pharmaceutical preparations, availability, Europe, stock-out, supply chain, medical need, pandemic, register

## Abstract

The study aimed to investigate medicine shortages of critical relevance in the pandemic. A total of 487 active substances for the treatment of COVID-19-related symptoms and therapeutically similar medicines were reviewed as to whether or not a shortage had been notified in Austria, Italy, and Spain for February 2020, March 2020, April 2020 (first wave of the pandemic), and, in comparison, in November 2021 (fourth wave). Publicly accessible shortage registers managed by the national regulatory authorities were consulted. For 48 active substances, a shortage was notified for at least one of the study months, mostly March and April 2020. Out of these 48 active substances, 30 had been explicitly recommended as COVID-19 therapy options. A total of 71% of the active substances with notified shortage concerned medicines labeled as essential by the World Health Organization. During the first wave, Spain and Italy had higher numbers of shortage notifications for the product sample, in terms of active substances as well as medicine presentations, than Austria. In November 2021, the number of shortage notifications for the studied substances reached lower levels in Austria and Spain. The study showed an increase in shortage notifications for COVID-19-relevant medicines in the first months of the pandemic.

## 1. Introduction

The COVID-19 pandemic has placed major constraints on pharmacists, who were confronted with several challenges, including managing medicine shortages [1,2].

Already before the outbreak of SARS-CoV-2, medicine shortages have become an issue of increasing concern globally [3,4,5,6]. In the last decade, a growing number of shortages was reported from several countries, including the United States of America, Canada, and European Union (EU) member countries [7,8,9,10,11,12,13,14,15,16].

Medicine shortages can have substantial negative impacts on patients and may result, in cases of lacking therapeutic substitutes, in prolonged hospital stays, delays and cancellations of medical procedures, ineffective treatment, and major incidences of adverse effects [17,18,19,20].

To mitigate medicine shortages, governments have introduced policy measures, such as requirements for pharmaceutical companies to notify upcoming shortages, supply reserves, parallel export bans, and stakeholder dialogues [21,22]. On a daily basis, pharmacists have been involved in managing shortages, for instance, by searching for alternative medicines, and these activities have considerably increased their workload [10,23,24,25].

Some countries (e.g., Austria, Italy, Spain) have introduced specific definitions for medicine shortages, frequently in relation to the reporting requirements for companies, and the national definitions slightly vary across countries [7,21,22]. In principle, as also stated by the European Medicines Agency, a shortage of medicine occurs when “supply does not meet demand at a national level where a ‘shortage’ as defined, allows for identification of current, impeding or anticipated disruption of supply of a medicinal product” [26].

Medicine shortages may result from a variety of root causes, such as production and quality problems, disruptions in the supply chain, regulatory and economic issues, unexpected events leading to increased medical needs, and planning errors so that demand is not met [7,16,27,28]. A pandemic such as COVID-19 is expected to negatively impact both supply and demand, as the supply chain may increasingly be exposed to vulnerabilities (disruptions in supply), and the need for medicines is likely to increase (growth in demand). Assuming these two developments, it can be hypothesized that the COVID-19 pandemic has led to increased shortages, specifically for medicines that are needed to treat symptoms of the infection.

Against this backdrop, the study aimed to investigate the extent of shortages of medicines used to treat COVID-19-related symptoms in three European countries (Austria, Italy, and Spain), in the early months of 2020 after the outbreak of SARS-CoV-2. As an additional research question, the extent to which shortages relating to the selected medicines were notified in these countries was studied for the fourth wave of the COVID-19 pandemic in autumn 2021 and to compare data from different periods.

## 2. Materials and Methods

The research question was developed around the timing of the declaration of the World Health Organization (WHO) of the COVID-19 pandemic (11 March 2020 [29]), and the methodological specifications for this study were defined in March 2020. For the follow-up research to investigate the situation during the fourth wave, the same methodology (e.g., with regard to countries, and products selected) was applied to allow a comparison across periods.

### 2.1. Country Selection

The selection of the countries was guided by the assumption that countries that were hit harder by the pandemic might likely experience higher need for medicines. We aimed to study if countries hit harder and thus with an increased need would also be confronted with more notifications of medicine shortages compared to countries that were hit less. We decided to choose two European countries which were hit hard by the COVID-19 pandemic, and, as a comparator, another European country that was hit less in the first wave.

Indicators to measure the impact of the pandemic in epidemiological terms include the number of cases, as well as death rates (excess mortality) in a normalized manner (i.e., per 100,000 inhabitants). Based on these data, Italy and Spain were selected because they were hit hard by the COVID-19 pandemic during the first wave and Austria was included as the country which was comparatively less affected at that time [30,31,32,33].

As a supplementary criterion, it was ensured that the selected countries had publicly accessible shortage registers.

### 2.2. Studied Time Periods

The research related to the three months of March, April, and May 2020, which covered most of the period of the first wave of the pandemic in Europe (according to epidemiologists the first wave came to an end at the end of June 2020 [32]), and to November 2021. The latter was chosen for follow-up research because cases had been rising again in European countries, with the fourth wave having started in mid-October 2021 [34], and we were interested in learning if in that restrained pandemic situation notifications of medicine shortages might reach similar dimensions as the ones experienced approximately one and a half years earlier or if vulnerabilities in the supply chain would be mitigated.

### 2.3. Product Selection

The medicines selected included those that had been recommended to treat COVID-19 symptoms according to the evidence available at the time of the study design (March 2020) and therapeutically similar medicines. As this was an early phase of the pandemic in European countries, the knowledge base on how to treat COVID-19 symptoms was yet limited and had been constantly evolving. Since we aimed to start the data retrieval as soon as possible after the outbreak of the pandemic, we had to base the product selection on guidance documents that were available at these early stages.

A two-stage approach was chosen for the product selection, based on a review of international evidence as a starting point, which was supplemented by guidance documents from the selected countries. However, regarding publications by supranational institutions, we could only identify a guidance document issued by the Pan American Health Organization (PAHO). That document, which had been published on 24 March 2020, recommended 37 active substances for the management of patients with suspected or confirmed COVID-19 diagnosis in intensive care units (ICUs) [35].

We identified COVID-19-related guidelines in the three study countries: the ICU therapy guideline for the treatment of patients with a SARS-CoV-2 infection of the Austrian Society for Anesthesiology, Reanimation and Intensive Medicine, published on 29 March 2020 [36], the guideline on therapeutic and support management for patients with COVID-19 coronavirus infection of the Italian Society of Infective and Tropical Diseases (a regularly updated document, we considered the “March 2020 edition”) [37], and a technical guidance document of the Spanish Ministry of Health for clinical management of COVID-19 hospital care, which was also regularly updated (we used the version published on 19 March 2020) [38].

We reviewed the guidelines from the three countries, focusing on identifying any further medicines that had not been covered by the PAHO guidance. In fact, 17 further active substances were mentioned in at least 1 of the country’s documents but had not been included in the PAHO guideline. For details on the active substances recommended in the four consulted guidance documents see Supplementary File S1.

The review of the guidance documents resulted in 54 active substances. Based on the assumption that other active substances of the same chemical subgroup (ATC-4 level), as defined by the Anatomic Therapeutic Chemical (ATC) classification of the WHO Collaborating Centre for Drug Statistics Methodology [39], might also be relevant with regard to the management of COVID-19 symptoms, we included all active substances of those chemical subgroups, independently of whether or not they have been indicated in the four consulted guidance documents. This amounted to a total of 487 ATC-5 codes (active substances or combinations of active substances). For details on all included active substances see Supplementary File S2.

A total of 90 of the selected active substances (18%) have been labeled as essential medicines by the WHO [40].

### 2.4. Data Sources and Analysis

To address medicine shortages, several European countries introduced medicine shortage registers, to which manufacturers and suppliers have been reporting shortages in advance. In Italy and Spain, manufacturers were mandated to notify upcoming shortages at any date in the study period; in Austria, this reporting obligation was introduced at the beginning of April 2020. Before, notifications were conducted on a voluntary basis [22]. In the three studied countries, the medicine agencies have been tasked to manage the shortage registers.

Since the medicine shortage registers are the official administrative databases of the regulatory agencies and serve as core sources for notifications of shortages, they were chosen for the search for shortage notifications for the selected active substances. In all three study countries (Austria, Italy, and Spain), these registers are publicly accessible [41,42,43].

The registers have been established at national levels, and their outline and content differ across the countries. Table 1 describes their characteristics.

For each of the selected active substances, it was checked whether or not a shortage had been reported to start in February 2020, March 2020, April 2020, and November 2021. Data retrieval was conducted from May to June 2020 and in December 2021. Medicines labeled as “partially unavailable” in the Austrian shortage register (next to the category of “full unavailability”) were included in the analysis.

Further information on the medicines for which a shortage had been notified was collected as far as included in the registers, e.g., causes or mitigation measures (Table 1). Guided by a piece of research that analyzed contents of shortage registers [44], a template for the data collection and further analysis was developed. In an Excel^®^ file, we collected key information on the medicines (e.g., trade name, active substance, ATC-5 code, dosage, pack size, route of administration) and the shortage (e.g., start and end of the shortage, reason for the shortage, availability of alternative medicines), where available.

The number of notifications on specific medicine presentations (in a defined dosage and route of administration) as well as the number of active substances for which a shortage (one or more notifications per active substance) had been notified were analyzed.

While the survey was based on the official shortage registers, we acknowledged that further information systems on shortages may exist. For instance, the Spanish pharmacists association managed a shortage catalog [45]. As a sensitivity analysis, data from that catalog were retrieved in 2020 (in 2021 this catalog was no longer accessible) to check for potential shortages that had not been reported to the agency’s register. However, the data from that catalog were not included in the analysis.

## 3. Results

In all three countries, medicine shortages were notified in the four study months. Overall, this concerned 48 of the studied 487 active substances (10%). Shortages were reported in 20 pharmacological groups (ATC-3 level according to the WHO ATC classification [39]).

A total of 30 of the 48 active substances for which a shortage was notified (63%) had been recommended as therapy options in 1 of the 4 consulted guidelines for the management of COVID-19 symptoms.

More than two out of three active substances for which a shortage was notified (thirty-three active substances) concerned essential medicines. This share of essential medicines among the substances for which a shortage had been notified (69%) was considerably higher than among the full study sample (18%).

Details are provided in Supplementary File S3.

There were differences in the number of shortage notifications between the countries and over time (Table 2). In terms of active substances, the number of newly reported shortages per month rose to 2-digit levels in Italy and Spain during March and April 2020 while for Austria this development only took place in April 2020. During the fourth wave in November 2021, Italy showed nearly the same high numbers of notified shortages of active substances, while respective figures for Spain and Austria were similar to dimensions in February and March 2020.

In the first wave of the COVID-19 pandemic, all three countries reported shortages of pantoprazole, rocuronium bromide, cisatracurium, and paracetamol.

For 54%, 35%, and 24% of the shortage notifications in Austria, Italy, and Spain, no information on the expected end of the shortage was included at the time of the data retrieval.

The reasons that were indicated most frequently in the Austrian and Italian registers included increased demand and manufacturing problems (no reasons indicated in the Spanish register). Changes were observed over time: for instance, 60% of the notifications for the studied active substances in the Italian dataset reported “increased demand” as a cause for the shortage during the first wave; the respective figure in November 2021 was 37%.

In 78% (Italy) and 88% (Spain, first wave) of the shortage notifications, a generic alternative was suggested as a mitigation measure. For detailed findings based on additional information included in the registers see Supplementary File S4.

The sensitivity analysis, for which data from the catalog of Spanish pharmacists was consulted, showed that shortages for three further active substances (lansoprazole, esomeprazole, and hydroxychloroquine) had been included in that data source, which had not been notified to the shortage register of the Spanish Medicines Agency.

## 4. Discussion

The study identified notifications of shortages of medicines recommended for the management of COVID-19-related symptoms in the first wave of the pandemic (February, March, and April 2020) and the fourth wave (November 2021). As the pandemic was gaining momentum in the spring of 2020, an increasing number of relevant medicines were notified to be unavailable. Shortages targeted important medication, including several essential medicines.

The findings of our study add to pieces of research in other countries with similar results. A study in Australia showed an increase in medicine shortages after the outbreak of SARS-CoV-2 in relation to pre-pandemic levels [46]. A study that analyzed sales and shortages of six substances in Portuguese community pharmacies from February to April 2020 also identified an increase in shortages during the first months of the pandemic [47]. An analysis of 121 cardiodiabetological medicines covering the years 2019 and 2020 showed overall limited availability in Polish pharmacies and a decrease in the availability of a few medicines after the outbreak of the COVID-19 pandemic [48]. A survey conducted from July to September 2020 with health professionals, mainly pharmacists, working in supply chain management in Saudi Arabia also highlighted increased shortages of medicines that were essential for their health system; reported substances with high levels of unavailability included tocilizumab, lopinavir/ritonavir, dexamethasone, and enoxaparin [49], for which shortages were notified in at least one of the three countries in our study.

The data of our research point to a growth in shortages of critical medicines, frequently those included in the WHO Model List of Essential Medicines [40], during the pandemic. This is in line with the findings of the studies in Portugal [47] and Saudi Arabia [49] and another piece of research on shortage notifications in four European countries, Australia, and the United States in 2021 which showed a concerning portion of essential medicines affected by shortages [50].

In all three studied countries, an increase in shortages (per medicine presentation and per active substance) for the product sample was observed in the first wave. In Austria this growth occurred in April 2020, thus one month later than in Italy and Spain. This may be attributable to the epidemiological situation as in the first wave Austria was hit less hard compared to Italy and Spain. But it may also result, to some extent, in differences in reporting. In Austria, notifications on expected and ongoing shortages by the pharmaceutical companies became mandatory on 1 April 2020; before that date it was voluntary. The legal change had been prepared long before the COVID-19 pandemic; upon implementation of this measure, Austria had the same legal framework as most other European countries that oblige pharmaceutical companies to notify shortages [22].

However, despite notification obligations, underreporting may be possible. Thus, the data presented in the study are to be interpreted as minimum figures. The sensitivity analysis conducted for this study, which consulted an additional source, confirmed this possibility, even if the difference between the two registers was minor.

Negative impacts of medicine shortages on patient safety and health outcomes are known [17,18,19,20]. Shortages identified in this study confirm this critical issue: for instance, midazolam and propofol were found to be out of stock in two studied countries during the first wave. This is of great concern as there was an urgent need for sedatives for COVID-19 patients who were intubated and placed on mechanical ventilation [51].

Mitigation measures for shortages include the search for alternative medicines, which meanwhile consumes an important share of the working time of pharmacists [23,52]. In the sample of this study, at least three out of four shortage notifications in the Italian and Spanish datasets (Spanish data only available for the first wave) indicated the existence of an alternative. However, for paracetamol, a high number of shortages was reported (45 shortage notifications solely in March and April 2020), which points to the possible unavailability of that substance.

It is difficult to determine the share of notified shortages that was attributable to increased demand, which was expressed as a concern in the literature given the growth in the use of and demand for narcotics, sedatives, and neuromuscular blockers [53], and of disruptions in manufacturing and distribution, which tend to increase in extraordinary situations such as a pandemic [54]. Our findings pointed to both factors: for instance, increased demand was more frequently mentioned in the first wave compared to the fourth wave in the Italian register, but capacity constraints in manufacturing and delays in production were also reported as reasons for the identified shortages in the Austrian and Italian registers. The latter causes confirm the vulnerability of the supply chains in the COVID-19 pandemic, especially during the first wave. However, the information on causes indicated in the registers was limited; it was not comparable across countries and even across months within a register, due to lack of standardization.

The findings of this study relating to earlier periods in the COVID-19 pandemic, which point to room for improvement of the shortage registers, also with regard to their cross-country comparability, are in line with learnings from the pandemic that have been expressed in the literature and the policy debate. Regulation (EU) 2022/123 [55], which became applicable in March 2022 and reinforced the role of the European Medicines Agency in crisis preparedness and management, can be seen as a political reaction. Based on this legislation, the European Medicines Agency has become responsible for monitoring medicine shortages that might lead to a crisis situation.

The study has some limitations. The product selection was based on state-of-the-art knowledge in March 2020 and concerned medicines for the treatment of COVID-19 symptoms, in particular in ICUs. In the meantime, knowledge has considerably improved on how to treat COVID-19 patients, and COVID-19 medications to treat the disease and not solely address symptoms have been authorized and marketed. All active substances of a chemical group (ATC-4) which included recommended medicines were reviewed but this does not imply that all these medicines would be appropriate for COVID-19 treatment. There can be gaps and errors in the shortage registers from which the information was retrieved, and comparability due to differences in national reporting requirements and the outline of the registers was limited. Given constraints regarding the possibilities to search for historic data in some registers (online databases), the study was designed to survey the shortage notifications in the respective months. Therefore, it is not known whether or not further medicines of the sample had already been unavailable months or years earlier. It is acknowledged that the choice of the “month” as time measurement is arbitrary since some shortages were notified on the first of a month and others on the 30th or 31st.

## 5. Conclusions

The study provides evidence of the occurrence of shortages of medicines needed to manage COVID-19 symptoms, and it suggests an increase during the first wave of the pandemic. These developments are likely attributable to growing demand as well as disruptions in the supply chain. At the same time, improvements in the reporting systems may also have led to an increase in shortage notifications.

By offering empirical data on medicine shortages related to COVID-19, the study adds to mostly anecdotal evidence that reported similar developments. Further research could provide additional insights, which could include repeating the study by adapting the product sample to account for updated clinical guidelines or by including further countries and later waves. Further research could also analyze whether or not identified shortages impacted the overall medicine provision for the studied substances. To do so, investigations based on additional data would be needed, i.e., to put identified shortages in relation to their use (e.g., based on sales or prescribing data). This would allow assessing if existing shortages could have been addressed through the use of alternative medicines, or if doctors and patients were confronted with a complete lack of appropriate medicines.

The research also highlighted gaps of important data in the registers and variations across the countries. As this limits the effectiveness of the registers as a tool to manage shortages, there is room for optimizing registers and working towards European harmonization.

Evidence on shortages of COVID-19-relevant medication can support policymakers who aim to optimize existing and planned policy measures to be better prepared for emergency situations including a global pandemic.

## Figures and Tables

**Table 1:** Characteristics of the shortage registers in the three selected countries in March 2020.

Country	Database Provider	Scope of Shortage Information	Update Frequency	Format and Accessibility	Start and End Date	Reason for Shortage	Mitigation Measures
Austria	Bundesamt für Sicherheit im Gesundheitswesen (BASG)	Unavailable and partially unavailable medicines as reported by the MAH	Constantly, published on the day after notification of the shortage	Publicly accessible web-based database	Start dates and expected and actual end dates are included	Included	Not included, but information is available whether, or not, an export ban has been imposed on the medicine in short supply
Italy	Agenzia Italiana del Farmaco (AIFA)	Temporary and permanent shortages as reported by the MAH	Every 2–3 weeks	Publicly accessible website that provides the latest updated Excel^®^ file	Start dates and expected end dates are included (no actual end dates)	Included	Included (e.g., alternative medicines, export ban)
Spain	Agencia Española de Medicamentos y Productos Sanitarios (AEMPS)	“Ongoing shortages” and “solved shortages” as reported by the MAH	Constantly, published on the day after notification of the shortage	Publicly accessible web-based database	Expected start dates and expected end dates are included (no actual end dates)	Not included	Included (e.g., alternative medicines, exceptional marketing)

MAH: Marketing authorization holder.

**Table 2:** Medicine shortage notifications for COVID-19 symptoms management and therapeutically similar medicines in the studied countries and months, per active substances and medicine presentation.

Country	February 2020		March 2020		April 2020		November 2021	
Austria	1 (1)	*Amoxicillin/* *clavulanic acid*	4 (5)	*Amoxicillin/* *clavulanic acid* Pantoprazole ***Oseltamivir*** Cisatracurium	14 (27)	Famotidine Pantoprazole Rabeprazole Esomeprazole *Granisetron* ***Sodium chloride*** ***Dexamethasone*** *Amoxicillin/clavulanic acid* *Combinations of penicillins* *Ciprofloxacin* Lamivudine/abacavir Rocuronium bromide ***Paracetamol*** ***Midazolam***	3 (4)	***Omeprazole*** ***Methylprednisolone*** *Ciprofloxacin*
Italy	3 (4)	Pantoprazole Esomeprazole Roxithromycin	13 (40)	Pantoprazole ***Methylprednisolone*** Roxithromycin *Ciprofloxacin* ***Lopinavir/ritonavir*** Darunavir/cobicistat ***Atracurium*** Rocuronium bromide Mivacurium chloride Cisatracurium ***Propofol*** ***Fentanyl*** ***Paracetamol***	14 (36)	Pantoprazole Esomeprazole ***Ondansetron*** ***Clarithromycin*** Lamivudine/abacavir **Tocilizumab** Rocuronium bromide Mivacurium chloride Cisatracurium ***Propofol*** ***Morphine*** ***Fentanyl*** ***Paracetamol*** ***Midazolam***	12 (19)	Pantoprazole Lansoprazole ***Heparin*** *Cefotaxime* ***Clarithromycin*** ***Azithromycin*** *Ciprofloxacin* Lamivudine/abacavir ***Lopinavir/ritonavir*** Rocuronium bromide ***Propofol*** ***Fentanyl***
Spain	6 (9)	**Omeprazole** Pantoprazole ***Ondansetron*** *Granisetron* ***Propofol*** ***Paracetamol***	17 (46)	**Omeprazole** ***Ondansetron*** Chlorhexidine *Hydrocortisone* ***Piperacilin/tazobactam*** ***Ceftazidime*** ***Clarithromycin*** ***Azithromycin*** ***Levofloxacin*** ***Moxifloxacin*** ***Atracurium*** Cisatracurium ***Propofol*** ***Morphine*** ***Fentanyl*** ***Paracetamol*** ***Haloperidol***	19 (67)	**Omeprazole** ***Ondansetron*** Enoxaparin Sodium bicarbonate Chlorhexidine ***Ethanol*** ***Methylprednisolone*** *Prednisone* *Hydrocortisone* *Amoxicillin/* *clavulanic acid* ***Piperacilin/tazobactam*** *Cefixime* ***Cefditoren*** ***Azithromycin*** ***Levofloxacin*** ***Suxamethonium*** Rocuronium bromide ***Propofol*** ***Paracetamol***	4 (6)	***Sodium chloride*** ***Moxifloxacin*** ***Vancomycin*** ***Paracetamol***

Data in brackets relate to the shortage notifications per medicine presentation in a given pharmaceutical form and dosage, as one active substance can be addressed by several notifications. A notification is counted in the month in which the shortage was notified to start. Active substances in bold indicate those recommended as therapy options for the treatment of COVID-19 symptoms (the others are active substances of the same ATC-4 groups); active substances in italic are those labeled as essential medicines by the WHO.

## Data Availability

The datasets supporting the conclusions of this article are included within the article and the Supplementary Materials.

## Supplementary Materials

The following supporting information can be downloaded at: https://www.mdpi.com/article/10.3390/pharmacy11040120/s1, Supplementary File S1: Medicines (listed by active substance) recommended in clinical guidelines as of March 2020 to treat COVID-19 symptoms; Supplementary File S2: Medicines (listed per ATC-5) recommended for the treatment of COVID-19 symptoms in guidelines as of March 2020 and further active substances of the same chemical group (ATC-4 group); Supplementary File S3: Shortage notifications for medicines to treat COVID-19 symptoms and further active substances of the same chemical group (ATC-4 group); Supplementary File S4: Background information retrieved from the shortage registers.
