# Supplementary material for: Shortages of Medicines to Treat COVID-19 Symptoms during the First Wave and Fourth Wave: Analysis of Notifications Reported to Registers in Austria, Italy, and Spain
Source: Pharmacy (Basel). 2023 Jul 21;11(4):120. doi: 10.3390/pharmacy11040120 (PMC10366777; doi:10.3390/pharmacy11040120)
Supplement: Supplementary file 1 [file pharmacy-11-00120-s001.zip › pharmacy-2437273-supplementary.pdf]

Supplementary Materials

Supplementary File S1.
Medicines (listed by active substance) recommended in clinical guidelines as of March 2020 to treat COVID-19 symptoms.

Active substance		Guideline			
**Name**	**ATC-5**	**PAHO**	**Austria**	**Italy**	**Spain**
ranitidine	A02BA02	√			
omeprazole	A02BC01	√			
atropine	A03BA01	√			
metoclopramide	A03FA01	√			
ondansetron	A04AA01	√			
heparin	B01AB01	√			
enoxaparin	B01AB05	√			
sodium chloride[1]	B05XA0	√	√		
potassium lactate	B05XA15	√			
norepinephrine	C01CA03	√	√		
dobutamine	C01CA07	√	√		√
epinephrine	C01CA24	√			
milrinone	C01CE02		√		
levosimendan	C01CX08		√		
chlorhexidine	D08AC02	√			
povidone-iodine	D08AG02	√			
ethanol	D08AX08	√			
dexamethasone	H02AB02			√	
methylprednisolone	H02AB04				√
hydrocortisone	H02AB09	√	√		√
amoxicillin / clavulanic acid	J01CR02	√			√
piperacillin / tazobactam	J01CR05	√			
ceftazidime	J01DD02	√			√
ceftriaxone	J01DD04	√			√
cefditoren	J01DD16				√
meropenem	J01DH02	√			
ceftaroline	J01DI02				√
clarithromycin	J01FA09				√
azithromycin	J01FA10	√			√
amikacin	J01GB06	√			
levofloxacin	J01MA12				√
moxifloxacin	J01MA14				√
vancomycin	J01XA01	√			
amphotericin B	J02AA01	√			
remdesivir	J05AB16		√	√	√
oseltamivir	J05AH02	√			
Lopinavir / ritonavir	J05AR10		√	√	√
favipiravir	J05AX27		√		
interferon beta-1b	L03AB08				√
anakinra	L04AC03				√
tocilizumab	L04AC07		√		√
suxamethonium	M03AB01	√			
atracurium	M03AC04	√			
propofol	N01AX10	√			

Active substance		Guideline			
**Name**	**ATC-5**	**PAHO**	**Austria**	**Italy**	**Spain**
morphine	N02AA01	√			
fentanyl	N02AB03	√			
paracetamol	N02BE01	√			
haloperidol	N05AD01	√			
lorazepam	N05BA06	√			
midazolam	N05CD08	√			
hydroxychloroquine	P01BA02		√	√	√
ipratropium bromide	R01AX03	√			
salbutamol	R03AC02	√			
oxygen	V03AN01	√	√	√	√

[1] In the PAHO recommendations and the Austrian guideline "saline solution" was indicated which can also include salt solutions (B05CB) such as sodium citrate, magnesium citrate, sodium bicarbonate and combinations.

Supplementary File S2:
Medicines (listed per ATC-5) recommended for the treatment of COVID-19 symptoms in guidelines as of March 2020 and further active substances of the same chemical group (ATC-4 group)

Active substance recommended for treatment of COVID-19 symptoms		ATC-4 code	Further substances in this ATC-4 group
**Name**	**ATC-5**		
ranitidine (EM)	A02BA02	A02BA	cimetidine<br>famotidine<br>nizatidine<br>niperotidine<br>roxatidine<br>ranitidine bismuth citrate<br>lafutidine<br>cimetidine, combinations<br>famotidine, combinations
omeprazole (EM)	A02BC01	A02BC	pantoprazole<br>rabeprazole<br>esomeprazole<br>dexlansoprazole<br>dexrabeprazole<br>vonoprazan<br>tegoprazan<br>lansoprazole, combinations<br>rabeprazole, combinations
atropine (EM)	A03BA01	A03BA	hyoscyamine<br>belladonna total alkaloids
metoclopramide (EM)	A03FA01	A03FA	cisapride<br>domperidone<br>bromopride<br>alizapride<br>clebopride<br>itopride<br>cinitapride<br>mosapride
ondansetron (EM)	A04AA01	A04AA	granisetron (EM)<br>tropisetron (EM)<br>dolasetron (EM)<br>palonosetron (EM)<br>palonosetron, combinations
heparin (EM)	B01AB01	B01AB	antithrombin III<br>dalteparin (EM)<br>nadroparin (EM)<br>parnaparin<br>reviparin<br>danaparoid<br>tinzaparin<br>sulodexide<br>bemiparin<br>heparin, combinations
enoxaparin (EM)	B01AB05		
sodium chloride (EM)[1]	B05XA03	B05XA	potassium chloride (EM)

Active substance recommended for treatment of COVID-19 symptoms		ATC-4 code	Further substances in this ATC-4 group
**Name**	**ATC-5**		
potassium lactate	B05XA15		sodium bicarbonate<br>ammonium chloride<br>magnesium sulfate (EM)<br>potassium phosphate, incl. combinations with other potassium salts<br>calcium chloride<br>sodium acetate<br>sodium phosphate<br>magnesium phosphate<br>magnesium chloride<br>zinc chloride<br>hydrochloric acid<br>sodium glycerophosphate<br>cardioplegia solutions<br>potassium acetate<br>zinc sulfate (EM)<br>combinations of electrolytes (EM)<br>electrolytes in combination with other drugs
norepinephrine<br>dobutamine<br>epinephrine (EM)	C01CA03<br>C01CA07<br>C01CA24	C01CA	etilefrine<br>isoprenaline<br>norepinephrine<br>dopamine (EM)<br>phenylephrine<br>oxedrine<br>metaraminol<br>methoxamine<br>mephentermine<br>dimetofrine<br>prenalterol<br>dopexamine<br>gepefrine<br>ibopamine<br>midodrine<br>octopamine<br>fenoldopam<br>cafedrine<br>arbutamine<br>theodrenaline<br>amezinium metilsulfate<br>ephedrine (EM)<br>droxidopa<br>combinations<br>etilefrine, combinations
milrinone	C01CE02	C01CE	amrinone<br>enoximone<br>bucladesine
levosimendan	C01CX08	C01CX	angiotensinamide<br>xamoterol<br>angiotensin II

Active substance recommended for treatment of COVID-19 symptoms		ATC-4 code	Further substances in this ATC-4 group
**Name**	**ATC-5**		
chlorhexidine (EM)	D08AC02	D08AC	dibrompropamidine<br>propamidine<br>hexamidine<br>polihexanide<br>chlorhexidine, combinations
povidone-iodine (EM)	D08AG02	D08AG	iodine/octylphenoxypolyglycolether<br>iodine (EM)<br>diiodohydroxypropane
ethanol (EM)	D08AX08	D08AX	hydrogen peroxide<br>eosin<br>propanol (EM)<br>tosylchloramide sodium<br>isopropanol<br>potassium permanganate (EM)<br>sodium hypochlorite<br>propanol, combinations
dexamethasone (EM)	H02AB02	H02AB	betamethasone (EM)<br>fluocortolone<br>paramethasone<br>prednisolone (EM)<br>prednisone (EM)<br>triamcinolone<br>cortisone<br>prednylidene<br>rimexolone<br>deflazacort<br>cloprednol<br>meprednisone<br>cortivazol
methylprednisolone (EM)	H02AB04		
hydrocortisone (EM)	H02AB09		
amoxicillin / clavulanic acid (EM)	J01CR02	J01CR	ampicillin and beta-lactamase inhibitor<br>amoxicillin and beta-lactamase inhibitor (other than clavulanic acid)<br>ticarcillin and beta-lactamase inhibitor<br>sultamicillin<br>piperacillin and beta-lactamase inhibitor other than tazobactam)<br>combinations of penicillins
piperacillin / tazobactam (EM)	J01CR05		

Active substance recommended for treatment of COVID-19 symptoms		ATC-4 code	Further substances in this ATC-4 group
Name	ATC-5		
ceftazidime (EM)	J01DD02	J01DD	cefotaxime (EM)<br>cefsulodin<br>cefmenoxime<br>latamoxef<br>ceftizoxime<br>cefixime (EM)<br>cefodizime<br>cefetamet<br>cefpiramide<br>cefoperazone<br>cefpodoxime<br>ceftibuten<br>cefdinir<br>cefcapene<br>cefteram<br>cefotaxime and beta-lactamase inhibitor<br>ceftazidime and beta-lactamase inhibitor (EM)<br>ceftriaxone, combinations<br>cefoperazone and beta-lactamase inhibitor<br>ceftriaxone and beta-lactamase inhibitor<br>cefpodoxime and beta-lactamase inhibitor
ceftriaxone (EM)	J01DD04		
cefditoren	J01DD16		
meropenem (EM)	J01DH02	J01DH	ertapenem<br>doripenem<br>biapenem<br>tebipenem pivoxil<br>imipenem and cilastatin<br>meropenem and vaborbactam<br>panipenem and betamipron<br>imipenem, cilastatin and relebactam
ceftaroline	J01DI02	J01DI	ceftobiprole medocaril<br>faropenem<br>cefiderocol (EM)<br>ceftolozane and beta-lactamase inhibitor
clarithromycin (EM)	J01FA09	J01FA	erythromycin (EM)<br>spiramycin<br>midecamycin<br>oleandomycin<br>roxithromycin<br>josamycin<br>troleandomycin<br>miocamycin<br>rokitamycin<br>dirithromycin<br>flurithromycin<br>telithromycin<br>solithromycin
azithromycin (EM)	J01FA10		

Active substance recommended for treatment of COVID-19 symptoms		ATC-4 code	Further substances in this ATC-4 group
**Name**	**ATC-5**		
amikacin (EM)	J01GB06	J01GB	tobramycin (EM)<br>gentamicin (EM)<br>kanamycin (EM)<br>neomycin<br>netilmicin (EM)<br>sisomicin<br>dibekacin<br>ribostamycin<br>isepamicin<br>arbekacin<br>bekanamycin<br>plazomicin (EM)
levofloxacin (EM)	J01MA12	J01MA	ofloxacin (EM)<br>ciprofloxacin (EM)<br>pefloxacin<br>enoxacin<br>temafloxacin<br>norfloxacin<br>lomefloxacin<br>fleroxacin<br>sparfloxacin<br>rufloxacin<br>grepafloxacin<br>trovafloxacin<br>gemifloxacin<br>gatifloxacin<br>prulifloxacin<br>pazufloxacin<br>garenoxacin<br>sitafloxacin<br>tosufloxacin<br>delafloxacin<br>levonadifloxacin<br>lascufloxacin
moxifloxacin (EM)	J01MA14		
vancomycin (EM)	J01XA01	J01XA	teicoplanin<br>telavancin<br>dalbavancin<br>oritavancin
amphotericin B (EM)	J02AA01	J02AA	hachimycin
remdesivir	J05AB16	J05AB	aciclovir (EM)<br>idoxuridine<br>vidarabine<br>ganciclovir<br>famciclovir<br>valaciclovir (EM)<br>cidofovir<br>penciclovir<br>valganciclovir (EM)<br>brivudine<br>brincidofovir

Active substance recommended for treatment of COVID-19 symptoms		ATC-4 code	Further substances in this ATC-4 group
**Name**	**ATC-5**		
oseltamivir (EM)	J05AH02	J05AH	zanamivir<br>peramivir<br>laninamivir
lopinavir / ritonavir (EM)	J05AR10	J05AR	zidovudine and lamivudine<br>lamivudine and abacavir (EM)<br>tenofovir disoproxil and emtricitabine<br>zidovudine, lamivudine and abacavir<br>zidovudine, lamivudine and nevirapine<br>emtricitabine, tenofovir disoproxil and efavirenz<br>stavudine, lamivudine and nevirapine<br>emtricitabine, tenofovir disoproxil and rilpivirine<br>emtricitabine, tenofovir disoproxil, elvitegravir and cobicistat<br>lamivudine, tenofovir disoproxil and efavirenz<br>lamivudine and tenofovir disoproxil<br>lamivudine, abacavir and dolutegravir<br>darunavir and cobicistat<br>atazanavir and cobicistat<br>lamivudine and raltegravir<br>emtricitabine and tenofovir alafenamide<br>emtricitabine, tenofovir alafenamide, elvitegravir and cobicistat<br>emtricitabine, tenofovir alafenamide and rilpivirine<br>emtricitabine, tenofovir alafenamide and bictegravir<br>dolutegravir and rilpivirine<br>emtricitabine, tenofovir alafenamide, darunavir and cobicistat<br>atazanavir and ritonavir (EM)<br>lamivudine, tenofovir disoproxil and doravirine<br>lamivudine and dolutegravir<br>darunavir and ritonavir<br>lamivudine, tenofovir disoproxil and dolutegravir
favipiravir	J05AX27	J05AX	moroxydine<br>lysozyme<br>inosine pranobex<br>pleconaril<br>enfuvirtide<br>maraviroc<br>maribavir<br>umifenovir<br>enisamium iodide<br>letermovir<br>tilorone<br>pentanedioic acid imidazolyl ethanamide<br>ibalizumab<br>tecovirimat<br>baloxavir marboxil<br>amenamevir<br>bulevirtide<br>fostemsavir<br>lenacapavir

Active substance recommended for treatment of COVID-19 symptoms		ATC-4 code	Further substances in this ATC-4 group
**Name**	**ATC-5**		
interferon beta-1b	L03AB08	L03AB	interferon alfa natural<br>interferon beta natural<br>interferon gamma<br>interferon alfa-2a<br>interferon alfa-2b<br>interferon alfa-n1<br>interferon beta-1a<br>interferon alfacon-1<br>peginterferon alfa-2b (EM)<br>peginterferon alfa-2a (EM)<br>albinterferon alfa-2b<br>peginterferon beta-1a<br>cepeginterferon alfa-2b<br>ropeginterferon alfa-2b<br>peginterferon alfacon-2<br>peginterferon alfa-2b, combinations<br>peginterferon alfa-2a, combinations
anakinra	L04AC03	L04AC	daclizumab<br>basiliximab<br>rilonacept<br>ustekinumab<br>canakinumab<br>briakinumab<br>secukinumab<br>siltuximab<br>brodalumab<br>ixekizumab<br>sarilumab<br>sirukumab<br>guselkumab<br>tildrakizumab<br>risankizumab<br>satralizumab<br>netakimab<br>bimekizumab<br>spesolimab
tocilizumab	L04AC07		
suxamethonium (EM)	M03AB01	M03AB	*no further active substances*
atracurium (EM)	M03AC04	M03AC	pancuronium<br>gallamine<br>vecuronium (EM)<br>hexafluronium<br>pipecuronium bromide<br>doxacurium chloride<br>fazadinium bromide<br>rocuronium bromide<br>mivacurium chloride<br>cisatracurium

Active substance recommended for treatment of COVID-19 symptoms		ATC-4 code	Further substances in this ATC-4 group
**Name**	**ATC-5**		
propofol (EM)	N01AX10	N01AX	ketamine (EM)<br>propanidid<br>alfaxalone<br>etomidate<br>sodium oxybate<br>nitrous oxide (EM)<br>esketamine<br>xenon<br>nitrous oxide, combinations
morphine (EM)	N02AA01	N02AA	opium<br>hydromorphone (EM)<br>nicomorphine<br>oxycodone (EM)<br>dihydrocodeine<br>papaveretum<br>oxymorphone<br>morphine, combinations<br>hydromorphone and naloxone<br>oxycodone and naloxone<br>oxycodone and naltrexone<br>dihydrocodeine, combinations<br>codeine, combinations excl. psycholeptics<br>codeine, combinations with psycholeptics
fentanyl (EM)	N02AB03	N02AB	ketobemidone<br>pethidine<br>pethidine, combinations excl. psycholeptics<br>pethidine, combinations with psycholeptics
paracetamol (EM)	N02BE01	N02BE	phenacetin<br>bucetin<br>propacetamol<br>paracetamol, combinations excl. psycholeptics<br>phenacetin, combinations excl. psycholeptics<br>bucetin, combinations excl. psycholeptics<br>paracetamol, combinations with psycholeptics<br>phenacetin, combinations with psycholeptics<br>bucetin, combinations with psycholeptics
haloperidol (EM)	N05AD01	N05AD	trifluperidol<br>melperone<br>moperone<br>pipamperone<br>bromperidol<br>benperidol<br>droperidol<br>fluanisone<br>lumateperone

Active substance recommended for treatment of COVID-19 symptoms		ATC-4 code	Further substances in this ATC-4 group
**Name**	**ATC-5**		
lorazepam (EM)	N05BA06	N05BA	diazepam (EM)<br>chlordiazepoxide<br>medazepam<br>oxazepam<br>potassium clorazepate<br>adinazolam<br>bromazepam<br>clobazam<br>ketazolam<br>prazepam<br>alprazolam<br>halazepam<br>pinazepam<br>camazepam<br>nordazepam<br>fludiazepam<br>ethyl loflazepate<br>etizolam<br>clotiazepam<br>cloxazolam<br>tofisopam<br>bentazepam<br>mexazolam<br>lorazepam, combinations
midazolam (EM)	N05CD08	N05CD	flurazepam<br>nitrazepam<br>flunitrazepam<br>estazolam<br>triazolam<br>lormetazepam<br>temazepam<br>brotizolam<br>quazepam<br>loprazolam<br>doxefazepam<br>cinolazepam<br>remimazolam<br>nimetazepam
ipratropium bromide (EM)	R01AX03	R01AX	calcium hexamine thiocyanate<br>retinol (EM)<br>ritiometan<br>mupirocin (EM)<br>hexamidine<br>framycetin<br>hyaluronic acid<br>various<br>combinations

Active substance recommended for treatment of COVID-19 symptoms		ATC-4 code	Further substances in this ATC-4 group
**Name**	**ATC-5**		
salbutamol (EM)	R03AC02	R03AC	fenoterol<br>rimiterol<br>hexoprenaline<br>isoetarin<br>pirbuterol<br>tretoquinol<br>carbuterol<br>tulobuterol<br>salmeterol (EM)<br>formoterol (EM)<br>clenbuterol<br>reproterol<br>procaterol<br>bitolterol<br>indacaterol<br>olodaterol
oxygen (EM)	V03AN01	V03AN	carbon dioxide<br>helium<br>nitrogen<br>medical air

EM = classified as essential medicine by the World Health Organization (https://apps.who.int/iris/rest/bit-streams/1145259/retrieve)

Supplementary File S3:
Shortage notifications for medicines to treat COVID-19 symptoms and further active substances of the same chemical group (ATC-4 group)

Medicine		Included in		Country	Shortage notifications			
Active substance	ATC-5	EML	GL		02 / 2020	03 / 2020	04 / 2020	11 / 2021
famotidine	A02BA03	N	N	AT	-	-	2	-
				IT	-	-	-	-
				ES	-	-	-	-
omeprazole	A02BC01	Y	Y	AT	-	-	-	2
				IT	-	-	-	-
				ES	1	3	2	-
pantoprazole	A02BC02	N	N	AT	-	1	1	-
				IT	2	1	1	1
				ES	1	-	-	-
lansoprazole	A02BC03	N	N	AT	-	-	-	-
				IT	-	-	-	1
				ES	-	-	-	-
rabeprazole	A02BC04	N	N	AT	-	-	1	-
				IT	-	-	-	-
				ES	-	-	-	-
esomeprazole	A02BC05	N	N	AT	-	-	1	-
				IT	1	-	1	-
				ES	-	-	-	-
ondansetron	A04AA01	Y	Y	AT	-	-	-	-
				IT	-	-	1	-
				ES	1	2	2	-
granisetron	A04AA02	Y	N	AT	-	-	1	-
				IT	-	-	-	-
				ES	2	-	-	-
heparin	B01AB01	Y	Y	AT	-	-	-	-
				IT	-	-	-	1
				ES	-	-	-	-
enoxaparin	B01AB05	N	N	AT	-	-	-	-
				IT	-	-	-	-
				ES	-	-	5	-
sodium bicarbonate	B05XA02	N	N	AT	-	-	-	-
				IT	-	-	-	-
				ES	-	-	1	-
sodium chloride	B05XA03	Y	Y	AT	-	-	1	-
				IT	-	-	-	-
				ES	-	-	-	1
chlorhexidine, combinations	D08AC52	N	N	AT	-	-	-	-
				IT	-	-	-	-
				ES	-	1	3	-
ethanol	D08AX08	Y	Y	AT	-	-	-	-
				IT	-	-	-	-
				ES	-	-	2	-
dexamethasone	H02AB02	Y	Y	AT	-	-	1	-
				IT	-	-	-	-
				ES	-	-	-	-

Medicine		Included in		Coun-try	Shortage notifications			
Active substance	ATC-5	EML	GL		02 / 2020	03 / 2020	04 / 2020	11 / 2021
methylpredni-solone	H02AB04	Y	Y	AT	-	-	-	1
				IT	-	2	-	-
				ES	-	-	1	-
prednisone	H02AB07	Y	N	AT	-	-	-	-
				IT	-	-	-	-
				ES	-	-	2	-
hydrocortisone	H02AB09	Y	N	AT	-	-	-	-
				IT	-	-	-	-
				ES	-	1	1	-
amoxicillin / clavulanic acid	J01CR02	Y	N	AT	1	2	1	-
				IT	-	-	-	-
				ES	-	-	1	-
piperacillin / tazobactam	J01CR05	Y	Y	AT	-	-	-	-
				IT	-	-	-	-
				ES	-	2	1	-
combinations of penicillins	J01CR50	Y	N	AT	-	-	4	-
				IT	-	-	-	-
				ES	-	-	-	-
cefotaxime	J01DD01	Y	N	AT	-	-	-	-
				IT	-	-	-	3
				ES	-	-	-	-
ceftazidime	J01DD02	Y	Y	AT	-	-	-	-
				IT	-	-	-	-
				ES	-	1	-	-
cefixime	J01DD08	Y	N	AT	-	-	-	-
				IT	-	-	-	-
				ES	-	-	1	-
cefditoren	J01DD16	N	Y	AT	-	-	-	-
				IT	-	-	-	-
				ES	-	-	1	-
roxithromycin	J01FA06	N	N	AT	-	-	-	-
				IT	1	1	-	-
				ES	-	-	-	-
clarithromycin	J01FA09	Y	Y	AT	-	-	-	-
				IT	-	-	2	4
				ES	-	2	-	-
azithromycin	J01FA10	Y	Y	AT	-	-	-	-
				IT	-	-	-	2
				ES	-	3	2	-
ciprofloxacin	J01MA02	Y	N	AT	-	-	1	1
				IT	-	1	-	1
				ES	-	-	-	-
levofloxacin	J01MA12	Y	Y	AT	-	-	-	-
				IT	-	-	-	-
				ES	-	2	1	-
moxifloxacin	J01MA14	Y	Y	AT	-	-	-	-
				IT	-	-	-	-
				ES	-	1	-	1

Medicine		Included in		Coun-try	Shortage notifications			
Active substance	ATC-5	EML	GL		02 / 2020	03 / 2020	04 / 2020	11 / 2021
vancomycin	J01XA01	Y	Y	AT				
				IT	-	-	-	-
				ES	-	-	-	1
oseltamivir	J05AH02	Y	Y	AT	-	1	-	-
				IT	-	-	-	-
				ES	-	-	-	-
lamivudine / abacavir	J05AR02	N	N	AT	-	-	1	-
				IT	-	-	1	1
				ES	-	-	-	-
lopinavir / ritonavir	J05AR10	Y	Y	AT	-	-	-	-
				IT	-	5	-	1
				ES	-	-	-	-
darunavir / cobicistat	J05AR14	N	N	AT	-	-	-	-
				IT	-	1	-	-
				ES	-	-	-	-
tocilizumab	L04AC07	N	Y	AT	-	-	-	-
				IT	-	-	1	-
				ES	-	-	-	-
suxamethonium	M03AB01	Y	Y	AT	-	-	-	-
				IT	-	-	-	-
				ES	-	-	1	-
atracurium	M03AC04	Y	Y	AT	-	-	-	-
				IT	-	4	-	-
				ES	-	1	-	-
rocuronium bromide	M03AC09	N	N	AT	-	-	1	-
				IT	-	5	1	1
				ES	-	-	1	-
mivacurium chloride	M03AC10	N	N	AT	-	-	-	-
				IT	-	1	1	-
				ES	-	-	-	-
cisatracurium	M03AC11	N	N	AT	-	1	-	-
				IT	-	3	5	-
				ES	-	4	-	-
propofol	N01AX10	Y	Y	AT	-	-	-	-
				IT	-	9	2	2
				ES	1	4	4	-
morphine	N02AA01	Y	Y	AT	-	-	-	-
				IT	-	-	2	-
				ES	-	1	-	-
fentanyl	N02AB03	Y	Y	AT	-	-	-	-
				IT	-	5	4	1
				ES	-	6	-	-
paracetamol	N02BE01	Y	Y	AT	-	-	7	-
				IT	-	2	9	-
				ES	3	10	35	3
haloperidol	N05AD01	Y	Y	AT	-	-	-	-
				IT	-	-	-	-
				ES	-	2	-	-

Medicine		Included in		Coun-try	Shortage notifications			
**Active substance**	**ATC-5**	**EML**	**GL**		**02 / 2020**	**03 / 2020**	**04 / 2020**	**11 / 2021**
midazolam	N05CD08	Y	Y	AT	-	-	4	-
				IT	-	-	5	-
				ES	-	-	-	-

AT = Austria, EML = WHO Model Essential Medicines List (information on whether, or not, a medicine has been in the WHO EML), ES = Spain, GL = guideline (information on whether, or not, a medicine has been included in at least one of the four reviewed guidelines), IT = Italy, N = no, Y = yes
Note: A notification is counted in the month in which the shortage was notified to start.

Supplementary File S4:
Background information retrieved from the shortage registers

Information on expected end dates as share of shortages notifications

End dates	Country	1st wave (02-04/2020)	4th wave (11/2021)	Total
No expected end date indicated	Austria	52%	75%	54%
	Italy	36%	32%	35%
	Spain	25%	17%	24%

Austria: 33 shortages notifications – 1st wave, 4 – 4th wave, 37 – total
Italy: 80 – 1st wave, 19 – 4th wave, 99 – total
Spain: 122 – 1st wave, 6 – 4th wave, 128 – total
Data do not sum up to 100% as further options (e.g. shortages resolved in later months) are possible.

Information on causes

**Austria**	**1st wave**	**4th wave**
**Causes for Austria**	**Total (n = 33)**	**Total (n = 4)**
Capacity constraints at the manufacturing site	24%	No specific cause was provided for the 4th wave; all included active substances targeted by a shortage notification indicated the reason “out of stock”
Delays in manufacturing	21%	
Increased demand	33%	
Other	9%	
Prioritisation of supply to other countries	3%	
Regulatory changes	3%	
Shortage of the active substance	6%	

”

**Italy - 1st wave**		**Italy - 4th wave**	
**Causes**	**n = 80**	**Causes**	**n = 19**
High demand: quota distribution	1%	High demand: discontinuous supplies	5%
Permanent marketing ceased	14%	Permanent marketing ceased	11%
Increased demand	60%	Increased demand	37%
Production problems	21%	Production problems	32%
Reduced availability: contingent distribution	4%	Temporary marketing ceased	16%

Italy: slightly different categories indicated for the 1st wave and 4th wave

Spain: Information not included in the register

Recommendation for alternatives

Austria: Information not included in the register

Italy:
Existence of generic alternatives for 76% (n = 80) in the 1st wave, 84% (n =19) in the 4th wave, 78% (n=99) in total

Spain:
Existence of generic alternatives for 88% (n = 122); no such information available for the 4th wave
